# Genome-sequence analysis of *Acinetobacter johnsonii* MB44 reveals potential nematode-virulent factors

**DOI:** 10.1186/s40064-016-2668-5

**Published:** 2016-07-04

**Authors:** Shijing Tian, Muhammad Ali, Li Xie, Lin Li

**Affiliations:** State Key Laboratory of Agricultural Microbiology, Huazhong Agricultural University, Wuhan, 430070 Hubei Province China; Biotechnology Program, Department of Environmental Sciences, COMSATS Institute of Information Technology, Abbottabad, Pakistan

**Keywords:** *Acinetobacter johnsonii*, Genome, Nematicidal activity

## Abstract

*Acinetobacter johnsonii* is generally recognized as a nonpathogenic bacterium although it is often found in hospital environments. However, a newly identified isolate of this species from a frost-plant-tissue sample, namely, *A. johnsonii* MB44, showed significant nematicidal activity against the model organism *Caenorhabditis elegans*. To expand our understanding of this bacterial species, we generated a draft genome sequence of MB44 and analyzed its genomic features related to nematicidal attributes. The 3.36 Mb long genome contains 3636 predicted protein-coding genes and 95 RNA genes (including 14 rRNA genes), with a G + C content of 41.37 %. Genomic analysis of the prediction of nematicidal proteins using the software MP3 revealed a total of 108 potential virulence proteins. Some of these proteins were homologous to the known virulent proteins identified from *Acinetobacter baumannii*, a pathogenic species of the genus *Acinetobacter*. These virulent proteins included the outer membrane protein A, the phospholipase D, and penicillin-binding protein 7/8. Moreover, one siderophore biosynthesis gene cluster and one capsular polysaccharide gene cluster, which were predicted to be important virulence factors for *C. elegans*, were identified in the MB44 genome. The current study demonstrated that *A. johnsonii* MB44, with its nematicidal activity, could be an opportunistic pathogen to animals.

**Electronic supplementary material:**

The online version of this article (doi:10.1186/s40064-016-2668-5) contains supplementary material, which is available to authorized users.

## Background

Bacterial species of the genus *Acinetobacter* are ubiquitous in nature and are usually found in the hospital environment; some of these species have been implicated in a variety of nosocomial infections (Bergogne-Berezin and Towner 1996). For instance, *Acinetobacter baumannii* is known as a global nosocomial pathogen for its ability to cause hospital outbreaks and develop antibiotic resistance (Dijkshoorn et al. 2007; Peleg et al. 2008); *A. pittii* and *A. nosocomialis* have been reported to be associated with human infections (Chuang et al. 2011; Wang et al. 2013). Certain *Acinetobacter* species are currently emphasized in discussions on pathogenicity and mechanisms of multidrug resistance. However, the species *A. johnsonii*, which was identified to encode an extended-spectrum β-lactamase that confers resistance against penicillins, cephalosporins, and monobactams (Zong 2014), has been scarcely reported to cause animal or human disease.

In this study, an *A. johnsonii* MB44 strain was isolated from a frost-plant-tissue sample in the process of screening for ice-nucleating bacteria (Li et al. 2012). Bioassay reveals the significant virulence of this strain against the model organism, *Caenorhabditis elegans*. To date, the human-pathogen *A. baumannii* and *A. nosocomialis* have been reported for their pathogenicity against *C. elegans* (Vila-Farres et al. 2015; Smith et al. 2004). Therefore, to identify the potential virulence factors and better understand the molecular mechanism of its ability to infect nematodes, we performed genome sequencing of *A. johnsonii* MB44. The genomic features and the potential nematode-virulent genes were reported herein.

## Methods

### Bacterial culture and genomic DNA preparation

A clonal population of *A. johnsonii* strain MB44 was derived from a single colony serially passaged three times. The bacterium was grown under incubation at 28 °C on Luria-Bertani (LB) agar plates (1.5 % agar) containing 0.5 % NaCl. Colonies were inoculated into 5 mL of LB medium with shaking at 28 °C for 24 h. Aliquots (250 μL) from the LB cultures were inoculated into 25 mL of LB broth in a 100 mL flask and incubated at 28 °C for 20 h. Cells were pelleted successively into one 1.5 mL centrifuge tube at 12,000 rpm. Genomic DNA was extracted using the Bacterial DNA Kit (GBCBIO), in accordance with the manufacturer’s protocol. DNA quality and quantity were determined with a Nanodrop spectrometer (Thermo Scientific, Wilmington, USA).

### Nematode toxicity bioassay

For pathogenicity assay, *C. elegans* strain N2 was maintained at NGM agar with *E. coli* OP50 as food source. Assay was conducted with age synchronized L4 stage worms. *A. johnsonii* MB44 was grown in LB broth for 24 h. The cells were collected, re-suspended, and diluted in M9 buffer to make desired initial concentrations (based on OD_600_). Assay was conducted in 96 well plate such as each well contained 150 µL of cell suspension, 5 µL of 8 mM 5-fluorodeoxyuridine (FUdR), 40 µL M9 buffer and 40–50 L4 worms. Killing of worms was observed after 72 h.

### Phylogenetic analysis

The 16S rRNA gene sequences of the reference strains used for phylogenetic analysis were obtained from GenBank database of the National Center for Biotechnology Information (NCBI) (Benson et al. 2015). To construct the phylogenetic tree, these sequences were collected and nucleotide sequence alignment was carried out using ClustalW (Thompson et al. 1994). The software MEGA v.5.05 (Tamura et al. 2011) was used to generate phylogenetic trees based on 16S rRNA genes under the neighbor-joining approach (Saitou and Nei 1987).

### Genome sequencing and assembly

The genome of MB44 was sequenced by a commercial service at Beijing BerryGenomics Co., Ltd. using the Illumina HiSeq 2000 platform. Genomic DNA was sequenced with the Illumina sequencing platform by the paired-end strategy (2 × 125 bp) and the details of library construction and sequencing can be found at the Illumina website, yielding 8,593,104 total reads and providing 137-fold coverage of the genome. ABySS v.1.3.7 (Simpson et al. 2009) was employed for sequence assembly and the optimal value of k-mer is 90. The final draft assembly contained 75 contigs and the total size of the genome is 3.36 Mb. Contigs were ordered based upon *Acinetobacter lwoffii* WJ10621 (Hu et al. 2011) as reference genome using Mauve (Darling et al. 2004). The circular genome of *A. johnsonii* MB44 was generated using Artemis (Rutherford et al. 2000).

### Genome annotation

Automated genome annotation was completed by the NCBI Prokaryotic Genome Annotation Pipeline (http://www.ncbi.nlm.nih.gov/genome/annotation_prok/). The coding sequences (CDSs) were predicted using software Glimmer v.3.02 (Delcher et al. 2007). The predicted CDSs were translated and used to search the NCBI non-redundant database, UniProt, and Clusters of Orthologous Groups (COG) databases. The whole genomic tRNAs were identified using tRNAscan-SE v.1.21 (Lowe and Eddy 1997), and rRNAs were found by RNAmmer v.1.2 Server (Lagesen et al. 2007). Genes with signal peptides were predicted by SignalP (Petersen et al. 2011). In addition, genes carrying trans-membrane helices were predicted by TMHMM (Moller et al. 2001); and CRISPR repeats were searched using CRISPRFinder (Grissa et al. 2007).

### Comparative genomics

The draft genome sequence of *A. johnsonii* MB44 (GenBank accession no. LBMO00000000.1) was compared with the available complete genome of *A. baumannii* AB307-0294 (GenBank: NC_011595.1) and *A. pittii* ANC4052 (GenBank: APQO00000000.1). A web server, named OrthoVenn (Wang et al. 2015), was adopted to identify orthologous clusters among the genomes of these species. The function of each orthologous cluster was deduced by BLASTP (Altschul et al. 1997) analysis against UniProt databases. A Venn diagram was created using the web application Venny (http://bioinfogp.cnb.csic.es/tools/venny/) with the orthologous cluster ID list. The average nucleotide identity among these species was calculated by the ANI (average nucleotide identity) calculator (Goris et al. 2007).

### Data deposition

This whole-genome shotgun project was deposited at DDBJ/EMBL/GenBank under the accession LBMO00000000. The version described in this paper is version LBMO01000000.

## Results and discussion

### Microbial features, classification, and nematode toxicity bioassay of MB44

**Fig. 1:**
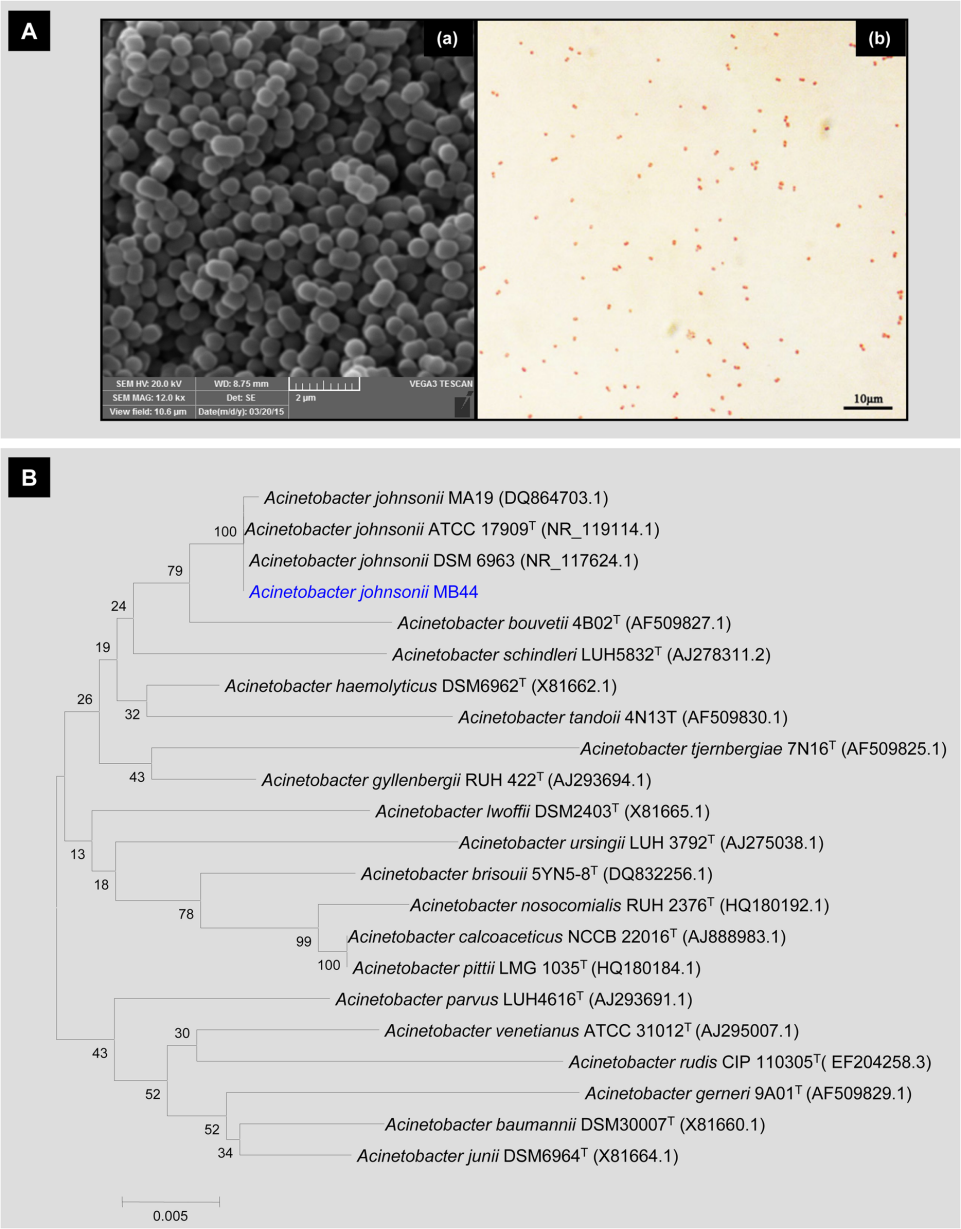
General characteristics of *A. johnsonii* MB44. **A** Micrographs of *A. johnsonii* MB44 cells in the exponential growth phase. (*a*) Cells under scanning electron microscopy; (*b*) Gram-stained cells under optical microscopy. **B** Neighbor-joining tree generated using MEGA 5 on the basis of 16S rRNA gene sequences. Bootstrap values are shown as percentages of 1000 replicates when these values are greater than 50 %. The *scale bar* represents 0.5 % substitution per nucleotide position

*Acinetobacter johnsonii* MB44 is a Gram-negative, non-sporulating, short, rod-shaped cells of 1.5–2.5 μm in length and 0.9–1.6 μm in width (Fig. 1A). MB44 cells are nonmotile and aerobic. The Kligler iron agar, nitrate reduction, oxidase reaction, and urea hydrolysis tests are negative, but the catalase and citrate utilization tests are positive. The microbe’s optimum growth temperature is 28–30 °C and no growth occurs at 37 °C or above. Lactose and glucose are fermented, but not for xylose. The DNA content (mol%) is 41.37 %. Prior to whole-genome sequencing, a 1434 bp 16S rRNA gene sequence was amplified by PCR using 27F (AGAGTTTGATCCTGGCTCAG) and 1492R (GGTTACCTTGTTACGACTT) then sequenced. Phylogenetic analysis based on the 16S rRNA gene of *A. johnsonii* MB44 is shown in Fig. 1B. As displayed, 99.65 % sequence identity to the 16S rRNA gene of *A. johnsonii* ATCC 17909^T^ was visualized.

**Fig. 2:**
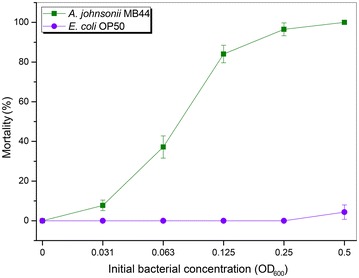
Bioassay of *A. johnsonii* cells against *C. elegans* L4 larva. *A. johnsonii* MB44 showed evident toxicity to *C. elegans* by the liquid killing assay. We used the fermentation product of *A. johnsonii* MB44 to test the strain’s toxicity against L4 nematodes in 96-well plates over six various initial bacterial concentrations (OD_600_) while comparing with the normal laboratory food *E. coli* OP50. *Error bars* represent the standard deviations from mean averages over three independent experiments

Unexpectedly, *A. johnsonii* MB44 exhibited remarkable nematicidal activity against *C. elegans*. As shown in Fig. 2, the MB44 suspension conferred over 80–100 % mortality to *C. elegans* after 72 h of host–pathogen interaction. This finding suggests the potential virulence of this strain to different animals. The strain could hence serve as a significant model microorganism for studying the fortuitous or potential bacterial pathogens in hospital environment.

### General features of the *A. johnsonii* MB44 genome sequence

**Fig. 3:**
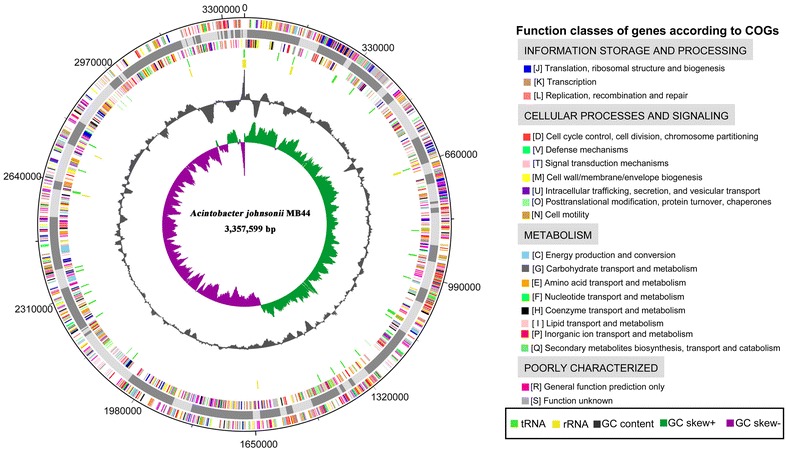
Circular representation of the *A. johnsonii* MB44 chromosome. The reference genome of *Acinetobacter lwoffii* WJ10621 was used to reorder the contigs of *A. johnsonii* MB44. The circular map was generated using Artemis. *Circles* from the center to the outside: GC skew (*spring green* and *purple*), GC content (*black*), rRNA (*yellow*), tRNA (*green*), genes on reverse strand colored by COG categories, 75 contigs in alternative grays, genes on forward strand colored by COG categories

The draft genome sequence consists of a chromosome of 3,357,599 bp in size. Moreover, 5.35 % of the predicted genes encoded signal peptides and 20.93 % of the genes possessed trans-membrane helices. A total of 58.0 % of CDSs could be assigned to the COG database. The distribution of genes into COG functional categories (Table 1; Fig. 3) shows that 39 predicted CDSs were involved in secondary metabolites biosynthesis and transport, such as siderophore synthesis, whereas 30 predicted CDSs were related to defense mechanisms, including several multidrug resistance efflux pumps.

**Table 1:** Number of genes associated with general COG functional categories

Category	Code^a^	Value	%Age^b^	Description
Information storage and processing	J	143	3.94	Translation, ribosomal structure and biogenesis
	K	118	3.25	Transcription
	L	183	5.05	Replication, recombination and repair
Cellular processes and signaling	D	25	0.69	Cell cycle control, Cell division, chromosome partitioning
	V	30	0.83	Defense mechanisms
	T	68	1.88	Signal transduction mechanisms
	M	156	4.30	Cell wall/membrane biogenesis
	N	25	0.69	Cell motility
	U	33	0.91	Intracellular trafficking and secretion
	O	92	2.54	Posttranslational modification, protein turnover, chaperones
Metabolism	C	139	3.83	Energy production and conversion
	G	73	2.01	Carbohydrate transport and metabolism
	E	179	4.94	Amino acid transport and metabolism
	F	52	1.43	Nucleotide transport and metabolism
	H	91	2.51	Coenzyme transport and metabolism
	I	134	3.70	Lipid transport and metabolism
	P	150	4.14	Inorganic ion transport and metabolism
	Q	39	1.08	Secondary metabolites biosynthesis, transport and catabolism
Poorly characterized	R	243	6.70	General function prediction only
	S	185	5.10	Function unknown

^a^Functional categories are represented according to the codes assigned by NCBI

^b^The total is based on the total number of predicted CDSs in the genome

### Comparative genomics

*Acinetobacter baumannii* is known as nosocomial pathogen for its ability to cause hospital outbreaks (Dijkshoorn et al. 2007; Peleg et al. 2008). *A. pittii* has been recently reported for its ability to cause disease in human (Wang et al. 2013). However, *A. johnsonii* was hardly reported to cause animal or human disease. Comparative genomics between *A. baumannii, A. pittii* and *A. johnsonii* will indicate the reason behind different abilities of these three strains to cause nosocomial infections at genome level. The general features of the genome sequence of *A. baumannii* AB307-0294, *A. pittii* ANC 4052, and *A. johnsonii* MB44 are shown in Table 2. The genome size of *A. johnsonii* MB44 was smaller than those of *A. baumannii* AB307-0294 and *A. pittii* ANC 4052. Moreover, the GC content of *A. johnsonii* MB44 was higher compared with those of *A. baumannii* AB307-0294 and *A. pittii* ANC 4052. Previously, ANI value between *A. johnsonii* MB44 and *A. johnsonii* ATCC 17909^T^ was calculated and it was found as 95.65 % (Tian et al. 2016). Furthermore, the ANI value between *A. johnsonii* MB44 and *A. baumannii* AB307-0294 was 79.72 %, whereas the ANI value between *A. johnsonii* MB44 and *A. johnsonii* XBB1 was 95.93 %, *A. johnsonii* MB44 and *A. johnsonii* SH046 was 95.81 %. This finding indicates that evolutionary relationship of *A. pittii* ANC 4052 and *A. baumannii* AB307-0294 are closer than that of *A. johnsonii* MB44.

**Table 2:** Comparison among the genome characteristics of *A. baumannii* AB307-0294, *A. pittii* ANC 4052, and *A. johnsonii* MB44

Feature	*A. johnsonii* MB44	*A. baumannii* AB307-0294	*A. pittii ANC* 4052
Finishing quality	Draft	Complete	Draft
Accession number	LBMO00000000	NC_011595	APQO00000000
Origin	Frost plant tissue	Blood	Blood
Genome size (bp)	3,357,599	3,760,981	3,95,339
G + C Content (mol%)	41.37	39.00	38.80
CDSs	3626	3513	3766
rRNA genes	14	18	18
tRNA genes	81	73	74

**Fig. 4:**
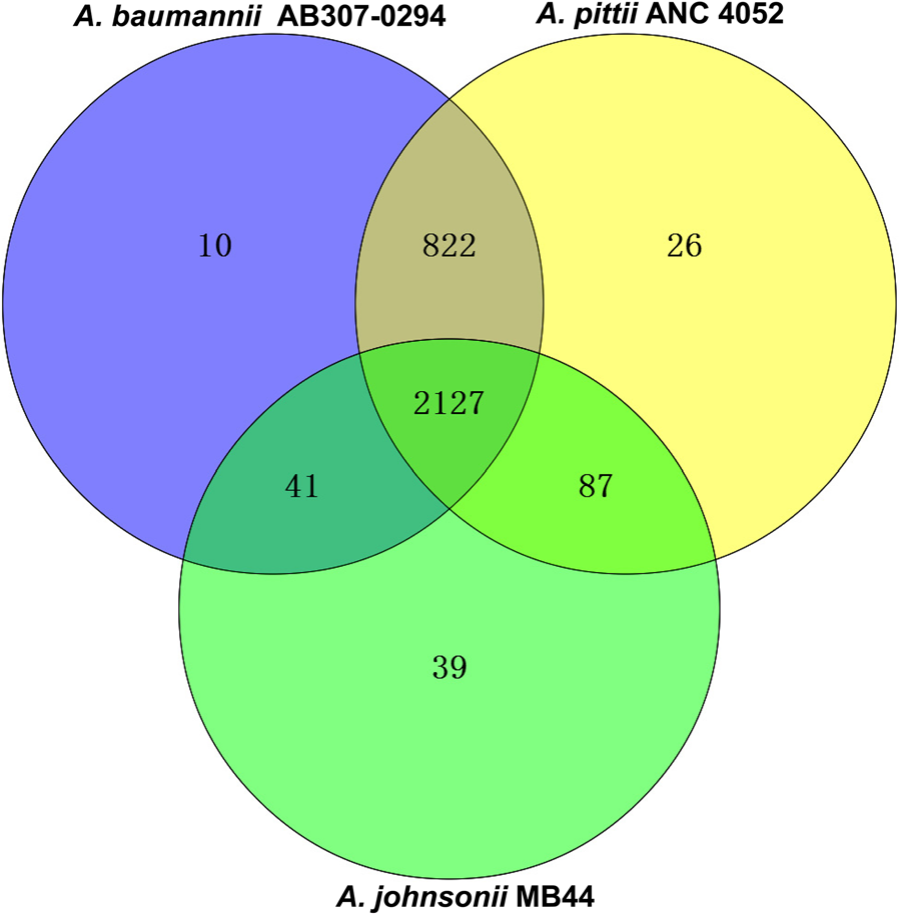
Visualization of the OrthoVenn output comparing the number of unique and/or shared orthologs of *A. baumannii* AB307-0294, *A. pittii* ANC 4052, and *A. johnsonii* MB44

The predicted proteome of *A. johnsonii* MB44 was assigned into orthologous clusters, along with the proteomes of *A. baumannii* AB307-0294 and *A. pittii* ANC 4052 to predict unique and/or shared characteristics among these species. As calculated within OrthoVenn, a total of 2127 putative orthologous proteins were shared among *A. baumannii* AB307-0294, *A. pittii* ANC 4052, and *A. johnsonii* MB44 (Fig. 4). *A. baumannii* AB307-0294 exhibited more shared orthologous proteins compared with *A. johnsonii* MB44 and the other two species. *A. baumannii* and *A. pittii* are both implicated in serious human infection. Hence, the genes shared by *A. baumannii* AB307-0294 and *A. pittii* ANC 4052 but were absent in *A. johnsonii* MB44 may be relevant in the two former species’ s ability to cause nosocomial infection.

### Genes predicted virulent to *C. elegans*

To predict the virulent proteins in the genomic data of *A. johnsonii* MB44, a software named MP3 (Gupta et al. 2014) was used to analyze the pathogenicity and comprehend the mechanism of pathogenesis by a hybrid Support Vector Machines (SVM) and Hidden Markov Model (HMM) approach. A total of 108 proteins of *A. johnsonii* MB44, with consistent predictions from both HMM and SVM, were classified as nematode-pathogenic (Additional file 1: Table S1). These putative virulent proteins were divided into four groups on the basis of mechanism of pathogenesis, particularly, structure/adhesion/colonization, invasion, secretion, and resistance (Roth 1988; Wu et al. 2008) (Table 3). Genes involved in the biosynthesis of fimbriae, LPS (lipopolysaccharide), porin, membrane protein, and phospholipase may promote pathogen adherence and invasion of host cells. These genes associated with Types I and II secretion systems may assist the transport of toxin. Transporters classified under the ATP-binding cassette superfamily, resistance–nodulation–division family, and major facilitator superfamily, which are associated with multidrug efflux pumps, may play important roles in antimicrobial resistance.

**Table 3:** Prediction of pathogenic proteins in *A. johnsonii* MB44 using MP3

Classification	Subclassification	Pathogenic proteins (HS)^a^		
Structure/adhesion/colonization	Fimbriae	AAU60_14105	AAU60_12470	AAU60_14060
		AAU60_14040	AAU60_14030	AAU60_13085
		AAU60_14155	AAU60_14035	
	LPS	AAU60_08620		
	Porin	AAU60_04175	AAU60_08060	
	Membrane protein	AAU60_03545	AAU60_00920	AAU60_00205
		AAU60_00470	AAU60_06630	AAU60_09350
		AAU60_01560	AAU60_04550	AAU60_13935
		AAU60_12465	AAU60_06330	
Invasion	Phospholipase	AAU60_09055	AAU60_12925	
Secretion	Type I secretion system	AAU60_04920	AAU60_04935	AAU60_13170
	Type II secretion system	AAU60_10120	AAU60_01405	AAU60_08540
		AAU60_10125	AAU60_01400	AAU60_08535
	ABC transporter	AAU60_06635	AAU60_00105	AAU60_07820
		AAU60_10860	AAU60_10765	AAU60_05600
		AAU60_04745	AAU60_09575	AAU60_15185
		AAU60_14255	AAU60_15880	AAU60_10855
	RND transporter	AAU60_15305	AAU60_13180	AAU60_06585
	MFS transporter	AAU60_15825	AAU60_07305	AAU60_08870
		AAU60_07850	AAU60_07470	AAU60_15415
Resistance	Drug/multi-drug resistance	AAU60_03375	AAU60_03380	AAU60_01105
		AAU60_00855		

^a^HS: predictions from both HMM (hidden markov model) and SVM (hybrid support vector machines) modules are in consensus

*Acinetobacter baumannii* is the most prevalent nosocomial pathogen of the genus *Acinetobacter*; thus, several recently identified virulence factors in *A. baumannii* (Cerqueira and Peleg 2011) have been found as homologous proteins in *A. johnsonii* MB44 (Table 4). Due to its known virulence factors against animal cells, *A. baumannii* was used as reference species for the prediction of nematicidal genes of MB44. Outer membrane protein A (OmpA) is a key virulence factor of *A. baumannii*, which localizes to the mitochondria and induces apoptosis of epithelial cells (Choi et al. 2005). Previous investigations revealed that OmpA can localize to the nucleus of eukaryotic cells and induce cytotoxicity (Choi et al. 2008a, b). The outer membrane protein (AAU60_12465) in *A. johnsonii* MB44 shared a 89 % amino acid sequence similarity with OmpA of *A. baumannii* ATCC 19606, inferring the former’s potential as a virulent protein. Moreover, phospholipase D (PLD) was demonstrated in *A. baumannii* to participate in the growth in human serum and epithelial cell invasion (Jacobs et al. 2010). Penicillin-binding protein 7/8 (PBP-7/8) is important for the survival of *A. baumannii* in a rat-soft-tissue infection model (Russo et al. 2009). The predicted phospholipase (AAU60_07280, AAU60_12565) and penicillin-binding protein in *A. johnsonii* MB44 (AAU60_01255) exhibited a high similarity to PLD and PBP-7/8. We therefore speculate that these genes may function as important virulence factors in *A. johnsonii* MB44.

**Table 4:** Potential virulent proteins in *A. johnsonii* MB44 and known homologous *A. baumannii* virulent proteins

Protein function	Gene accession number	Major motif	Virulent protein	Microorganism	Amino acid similarity (%)
Penicillin-binding protein 7/8	AAU60_01255	Transpeptidase superfamily	PBP-7/8	*A. baumannii* strain 307-0294	82
Phospholipase D	AAU60_07280	PLDc_SF superfamily	PLD	*A. baumannii* strain 98-37-09	62
	AAU60_12565	PLDc_SF superfamily	PLD	*A. baumannii* strain 98-37-09	85
Outer membrane protein A	AAU60_12465	OmpA_C-like superfamily	OmpA	*A. baumannii* ATCC 19606	89

### Putative genes involved in siderophore biosynthesis and transport

**Fig. 5:**
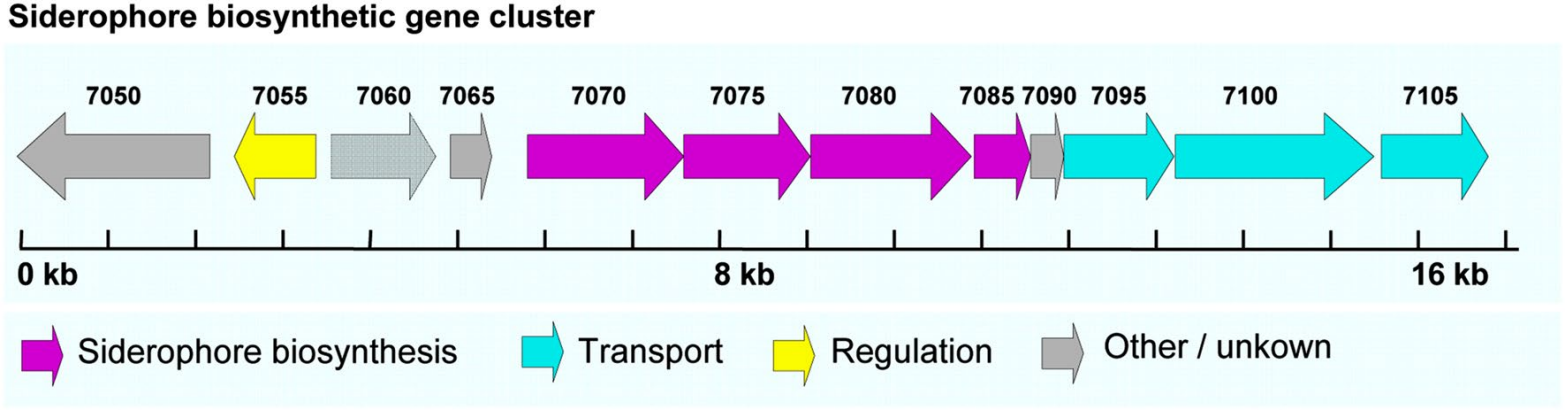
Gene organization of the putative siderophore biosynthesis gene cluster found in *A. johnsonii* MB44 genome. The putative 16.76 kb gene cluster carries 12 open reading frames (AAU60_07070–AAU60_07085). The proteins encoded by siderophore biosynthesis genes (*colored magenta*) of the siderophore biosynthetic gene cluster showing high identity (63–79 %) to an identified siderophore gene cluster of *A. haemolyticus* ATCC 17906^T^

Recent studies demonstrated that iron acquisition systems are important virulence factors in some pathogenic bacteria; such pathogens employed siderophores to acquire growth-essential iron from the host (Schaible and Kaufmann 2004; Weinberg 2009). The iron-binding siderophore produced by *Pseudomonas aeruginosa* is found to be a key virulence factor in disrupting mitochondrial and iron homeostasis in *C. elegans* (Kirienko et al. 2015). The ability to synthesize siderophores was believed to potentially affect the virulence of *A. johnsonii* MB44 against *C. elegans*. In the draft genome of *A. johnsonii* MB44, four secondary metabolite gene clusters were found using the antiSMASH pipeline (Weber et al. 2015), including one siderophore cluster (AAU60_07050–AAU60_07105). The siderophore gene cluster (Fig. 5) consists of 16,761 bp of nucleotide sequence, putatively containing four siderophore biosynthesis genes (AAU60_07070–AAU60_07085). Protein products of these four genes show high identity (63–79 %) to a recognized siderophore gene cluster of *A. haemolyticus* ATCC 17906^T^ (Funahashi et al. 2013). In the putative siderophore cluster of *A. johnsonii* MB44, three genes (AAU60_7095–AAU60_7105) were involved in the transport of siderophore. AAU60_7100 encodes a TonB-dependent siderophore receptor. The products of AAU60_7095 and AAU60_7105, which belong to the major facilitator superfamily of proteins, act as siderophore transporter and exporter, respectively.

### Capsular polysaccharide gene cluster

**Fig. 6:**
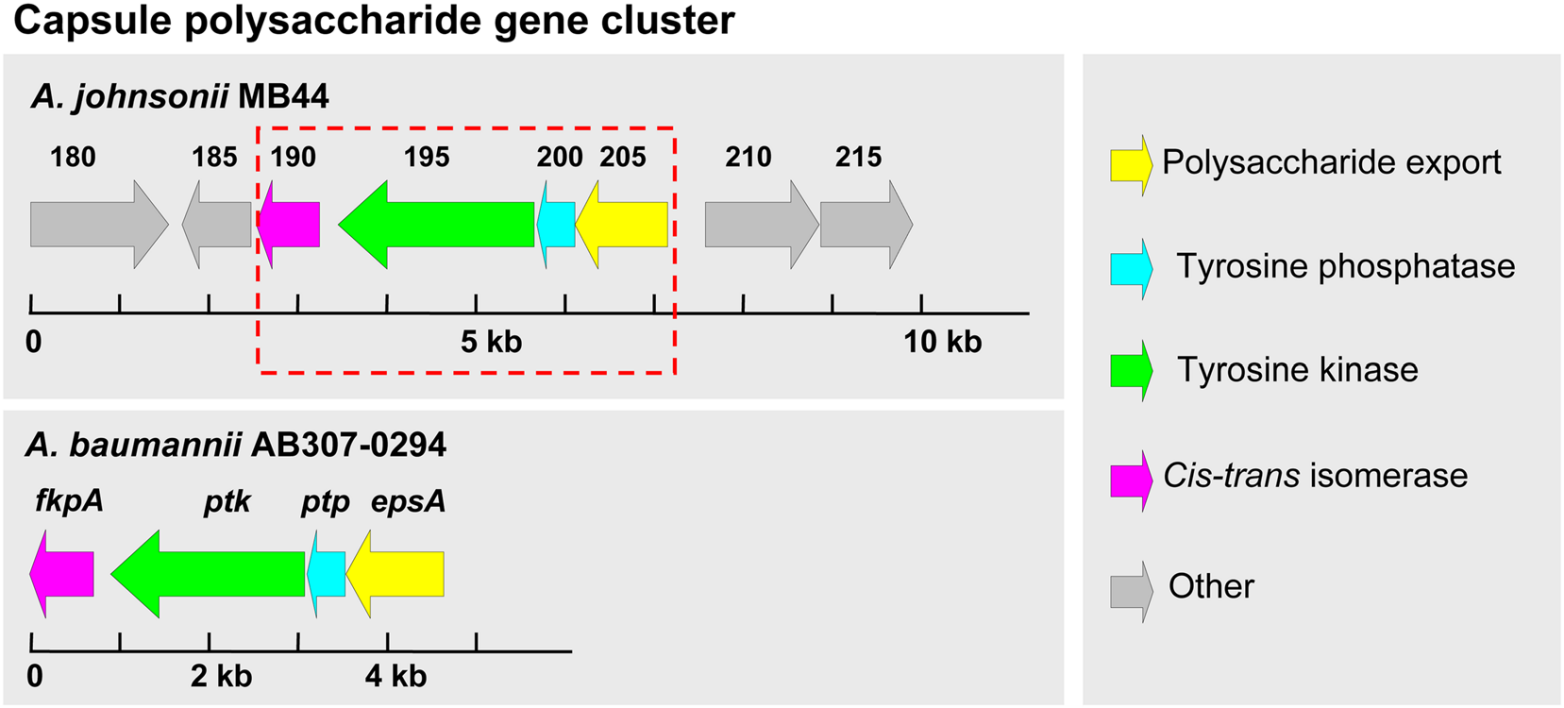
Genetic organization and conservation of the capsule polysaccharide cluster found in the *A. johnsonii* MB44 genome. The capsular cluster of *A. johnsonii* MB44 consists of four open reading frames (AAU60_00190–AAU60_00250). The identified capsular cluster of *A. baumannii* AB307-0294 is shown for comparison. The proteins encoded by the capsular cluster shown in the *red dashed* border exhibit high similarity (78–80 %) to the capsular gene cluster of *A. baumannii* AB307-0294

Capsules are important virulence factors that enable pathogenic bacteria to avoid the host defense mechanisms by their antiphagocytic ability (Heumann and Roger 2002). The K1 capsular polysaccharide from *A. baumannii* AB307-0294 has been demonstrated in a rat-soft-tissue infection model as a major virulence factor (Russo et al. 2010). The capsular cluster of *A. baumannii* AB307-0294 consists of 10 kb of sequence containing four genes (*fkpA*, *ptk*, *ptp*, and *epsA*), facilitating the polymerization and transport of capsular polysaccharide. These genes were aligned against the genome sequence of *A. johnsonii* MB44 with BLAST. Subsequently, four genes (AAU60_00190–AAU60_00205), with amino-acid sequences exhibiting high similarity (78–80 %) to the capsular gene cluster of *A. baumannii* AB307-0294, were found to constitute the capsular cluster of *A. johnsonii* MB44 (Fig. 6; Table 5). Therefore, these four genes were putatively involved in capsular polysaccharide polymerization and transport of *A. johnsonii* MB44. Prior studies suggested that insertions in genes involved in the capsular polysaccharide biosynthesis of *Staphylococcusaureus* reduce *C. elegans* deaths (Bae et al. 2004). Accordingly, we speculate that capsular polysaccharide may be a virulence factor of *A. johnsonii* MB44 involved in *C. elegans* lethal infection.

**Table 5:** Summary of homology searches for the open reading frames found in the putative capsule cluster of *A. johnsonii* MB44

ORF (aa) (*A. johnsonii* MB44)	Homologous protein (aa) (*A. baumannii* AB307-0294)	Identity/similarity (%) (aa overlap)	Function predicted
AAU60_00190 (234)	FkpA (240)	66/78 (242)	Peptidyl-prolyl *cis*-*trans* isomerase
AAU60_00195 (731)	Ptp (727)	65/80 (714)	Protein tyrosine kinase
AAU60_00200 (142)	Ptp (142)	64/80	Tyrosine phosphatase
AAU60_00205 (344)	EpsA (366)	63/79	Polysaccharide export outer membrane protein

## Conclusions

In this study, we presented a whole-genome analysis of *A. johnsonii* MB44 to identify its potential virulence factors against *C. elegans*. The MB44 genome contained 108 virulent proteins predicted by MP3, and four proteins showed high identity to the known virulent proteins in the pathogenic *A. baumannii.* Furthermore, one siderophore biosynthesis gene cluster and one capsular polysaccharide gene cluster were identified, which were relevant to nematicidal activity of pathogenic bacteria. The current study demonstrated that *A. johnsonii*, which was generally recognized as a nonpathogenic bacterium, could be an opportunistic pathogen to animals.

## Additional file

10.1186/s40064-016-2668-5 The predicted nematode-virulent genes in *A johnsonii* genome.
