# Supplementary material for: Genome-sequence analysis of Acinetobacter johnsonii MB44 reveals potential nematode-virulent factors
Source: Springerplus. 2016 Jul 4;5(1):986. doi: 10.1186/s40064-016-2668-5 (PMC4932006; doi:10.1186/s40064-016-2668-5)
Supplement: Supplementary file 1 — 10.1186/s40064-016-2668-5 The predicted nematode-virulent genes in A johnsonii genome. [file 40064_2016_2668_MOESM1_ESM.doc]

**Table S1**. The predicted nematode-virulent genes in *A johnsonii* genome.

| **Gene accession number** | **Deduced function** | **Number of amino acid** | **Homologous protein (GenBank accession number)** | **Host bacterial strain** | **Sequence identity (%)** |
| --- | --- | --- | --- | --- | --- |
| AAU60_00105 | Sulfonate ABC transporter substrate-binding protein | 331 | WP_010325546.1 | *Acinetobacter lwoffii* | 94% |
| AAU60_00205 | Membrane protein | 344 | WP_046760473.1 | *Acinetobacter* sp. Ag2 | 84% |
| AAU60_00470 | Membrane protein | 213 | WP_016166717.1 | *Acinetobacter tandoii* | 96% |
| AAU60_00755 | Glutathione peroxidase | 181 | WP_005173845.1 | *Acinetobacter* sp. NIPH 713 | 89% |
| AAU60_00855 | EmrB/QacA subfamily drug resistance transporter | 464 | WP_010325672.1 | *Acinetobacter lwoffii* | 98% |
| AAU60_00920 | Membrane protein | 441 | WP_042892338.1 | *Acinetobacter junii* | 97% |
| AAU60_00990 | transporter | 399 | WP_004708966.1 | *Acinetobacter nosocomialis* | 96% |
| AAU60_01105 | Multidrug transporter | 493 | WP_004777769.1 | *Acinetobacter* sp. CIP 102082 | 98% |
| AAU60_01255 | D-alanyl-D-alanine endopeptidase | 343 | WP_034587569.1 | *Acinetobacter* sp. HR7 | 78% |
| AAU60_01335 | RND transporter | 442 | WP_045796504.1 | *Acinetobacter indicus* | 77% |
| AAU60_01400 | General secretion pathway protein | 274 | WP_010325725.1 | *Acinetobacter lwoffii* | 99% |
| AAU60_01405 | General secretion pathway protein GspD | 786 | WP_004650662.1 | *Acinetobacter bohemicus* | 79% |
| AAU60_01560 | Membrane protein | 195 | WP_035265961.1 | *Acinetobacter* sp. Ver3 | 84% |
| AAU60_01900 | β-lactamase | 432 | WP_010325789.1 | *Acinetobacter lwoffii* | 99% |
| AAU60_03020 | Transporter | 421 | WP_004649809.1 | *Acinetobacter bohemicus* | 85% |
| AAU60_03255 | Peptidase | 277 | WP_034676199.1 | *Acinetobacter baumannii* | 87% |
| AAU60_03375 | Multidrug transporter | 105 | WP_045795531.1 | *Acinetobacter indicus* | 96% |
| AAU60_03380 | Multidrug transporter | 105 | WP_035268289.1 | *Acinetobacter* sp. Ver3 | 89% |
| AAU60_03545 | Membrane protein | 216 | WP_008305529.1 | *Acinetobacter* sp. HA | 95% |
| AAU60_04175 | Porin | 374 | WP_044112846.1 | *Acinetobacter* sp. MII | 80% |
| AAU60_04320 | Hydrolase | 324 | WP_004648055.1 | *Acinetobacter bohemicus* | 95% |
| AAU60_04370 | C4-dicarboxylate ABC transporter substrate-binding protein | 336 | WP_054581326.1 | *Acinetobacter* sp. 114 | 82% |
| AAU60_04440 | Chemotaxis protein | 690 | WP_005008060.1 | *Acinetobacter bouvetii* | 88% |
| AAU60_04550 | Membrane protein | 724 | WP_004648155.1 | *Acinetobacter bohemicus* | 86% |
| AAU60_04745 | ABC transporter permease | 829 | WP_010326261.1 | *Acinetobacter lwoffii* | 97% |
| AAU60_04920 | Type I secretion protein | 721 | WP_052755120.1 | *Acinetobacter baumannii* | 71% |
| AAU60_04925 | RND transporter | 504 | WP_005134196.1 | *Acinetobacter baumannii* | 94% |
| AAU60_04935 | Secretion protein HlyD | 395 | WP_005134206.1 | *Acinetobacter baumannii* | 90% |
| AAU60_05025 | Lactoylglutathione lyase | 126 | WP_010326302.1 | *Acinetobacter lwoffii* | 98% |
| AAU60_05110 | TonB-dependent receptor | 828 | WP_010326320.1 | *Acinetobacter lwoffii* | 99% |
| AAU60_05245 | D-alanyl-D-alanine carboxypeptidase | 382 | WP_010326342.1 | *Acinetobacter lwoffii* | 99% |
| AAU60_05300 | Hypothetical protein | 227 | WP_004651400.1 | *Acinetobacter bohemicus* | 85% |
| AAU60_05330 | Peptide transporter | 613 | WP_010326358.1 | *Acinetobacter lwoffii* | 97% |
| AAU60_05600 | Cell division protein FtsX | 411 | WP_004648319.1 | *Acinetobacter bohemicus* | 92% |
| AAU60_05785 | Peroxiredoxin | 152 | WP_010326425.1 | *Acinetobacter lwoffii* | 98% |
| AAU60_06080 | Hypothetical protein | 117 | WP_053580001.1 | *Acinetobacter* sp. TTH0-4 | 67% |
| AAU60_06145 | Hypothetical protein | 88 | WP_005026781.1 | *Acinetobacter radioresistens* | 33% |
| AAU60_06180 | Lysozyme | 168 | WP_016167360.1 | *Acinetobacter tandoii* | 84% |
| AAU60_06330 | Membrane protein | 194 | WP_010326453.1 | *Acinetobacter lwoffii* | 98% |
| AAU60_06520 | ATPase AAA | 318 | WP_010326473.1 | *Acinetobacter lwoffii* | 98% |
| AAU60_06585 | RND transporter | 455 | WP_046206365.1 | *Acinetobacter radioresistens* | 99% |
| AAU60_06630 | Membrane protein | 376 | WP_010326544.1 | *Acinetobacter lwoffii* | 96% |
| AAU60_06635 | Antibiotic ABC transporter permease | 371 | WP_029575002.1 | *Acinetobacter lwoffii* | 98% |
| AAU60_07305 | MFS transporter | 392 | WP_004649423.1 | *Acinetobacter bohemicus* | 75% |
| AAU60_07470 | MFS transporter | 455 | WP_010326682.1 | *Acinetobacter lwoffii* | 99% |
| AAU60_07525 | Cation transporter | 440 | WP_004976952.1 | *Acinetobacter towneri* | 98% |
| AAU60_07750 | D-Ala-D-Ala carboxypeptidase | 323 | WP_010326732.1 | *Acinetobacter lwoffii* | 97% |
| AAU60_07820 | ABC transporter substrate-binding protein | 340 | WP_010326745.1 | *Acinetobacter lwoffii* | 99% |
| AAU60_07850 | MFS transporter | 384 | WP_029575031.1 | *Acinetobacter lwoffii* | 99% |
| AAU60_08060 | Porin | 350 | WP_010326783.1 | *Acinetobacter lwoffii* | 98% |
| AAU60_08385 | quaternary ammonium transporter | 114 | WP_034696520.1 | *Acinetobacter bohemicus* | 86% |
| AAU60_08535 | General secretion pathway protein GspJ | 206 | WP_010326850.1 | *Acinetobacter lwoffii* | 98% |
| AAU60_08540 | General secretion pathway protein GspI | 133 | WP_004648758.1 | *Acinetobacter bohemicus* | 78% |
| AAU60_08620 | LPS biosynthesis protein | 819 | WP_010326862.1 | *Acinetobacter lwoffii* | 99% |
| AAU60_08870 | MFS transporter | 381 | WP_010326895.1 | *Acinetobacter lwoffii* | 98% |
| AAU60_08910 | D-alanyl-D-alanine carboxypeptidase | 436 | WP_010326934.1 | *Acinetobacter lwoffii* | 99% |
| AAU60_08960 | IroE protein | 308 | WP_005009620.1 | *Acinetobacter bouvetii* | 69% |
| AAU60_09055 | Phospholipase | 381 | WP_010326957.1 | *Acinetobacter lwoffii* | 96% |
| AAU60_09160 | Transporter | 424 | WP_048881938.1 | *Acinetobacter* sp. VT 511 | 98% |
| AAU60_09350 | Membrane protein | 832 | WP_029575049.1 | *Acinetobacter lwoffii* | 94% |
| AAU60_09505 | Acyltransferase | 439 | WP_010327012.1 | *Acinetobacter lwoffii* | 98% |
| AAU60_09575 | Sulfonate ABC transporter substrate-binding protein | 319 | WP_010327025.1 | *Acinetobacter lwoffii* | 98% |
| AAU60_09595 | Hypothetical | 141 | WP_010327029.1 | *Acinetobacter lwoffii* | 96% |
| AAU60_09600 | Hypothetical | 175 | WP_042089673.1 | *Acinetobacter bereziniae* | 73% |
| AAU60_09630 | RDD family protein | 255 | WP_010327034.1 | *Acinetobacter lwoffii* | 96% |
| AAU60_10120 | Type II secretion system protein M | 159 | WP_010327096.1 | *Acinetobacter lwoffii* | 97% |
| AAU60_10125 | General secretion pathway protein GspL | 379 | WP_010327097.1 | *Acinetobacter lwoffii* | 97% |
| AAU60_10765 | ABC transporter permease | 257 | WP_019836575.1 | *Acinetobacter* sp. MDS7A | 95% |
| AAU60_10845 | Transporter | 495 | WP_050796978.1 | *Acinetobacter lwoffii* | 93% |
| AAU60_10855 | Multidrug ABC transporter permease | 376 | WP_010327219.1 | *Acinetobacter lwoffii* | 98% |
| AAU60_10860 | Multidrug ABC transporter permease | 362 | WP_010327220.1 | *Acinetobacter lwoffii* | 97% |
| AAU60_11300 | Hypothetical | 148 | WP_053578436.1 | *Acinetobacter* sp. TTH0-4 | 68% |
| AAU60_11545 | Hypothetical | 569 | WP_029575087.1 | *Acinetobacter lwoffii* | 99% |
| AAU60_11755 | Membrane_protein | 232 | WP_019837053.1 | *Acinetobacter* sp. MDS7A | 83% |
| AAU60_11805 | Outer membrane efflux protein OprM | 485 | WP_010327336.1 | *Acinetobacter lwoffii* | 99% |
| AAU60_12020 | Peptidoglycan-binding protein LysM | 157 | WP_019837735.1 | *Acinetobacter* sp. MDS7A | 92% |
| AAU60_12105 | Heme-binding protein | 594 | WP_010327376.1 | *Acinetobacter lwoffii* | 99% |
| AAU60_12165 | Bacterioferritin comigratory protein | 189 | WP_029575099.1 | *Acinetobacter lwoffii* | 99% |
| AAU60_12465 | Membrane protein | 340 | WP_010327439.1 | *Acinetobacter lwoffii* | 99% |
| AAU60_12470 | Fimbrial biogenesis protein FimT | 174 | WP_010327440.1 | *Acinetobacter lwoffii* | 94% |
| AAU60_12480 | Glyoxalase | 150 | WP_010327441.1 | *Acinetobacter lwoffii* | 97% |
| AAU60_12925 | Zinc dependent phospholipase C | 254 | WP_010327504.1 | *Acinetobacter lwoffii* | 97% |
| AAU60_13085 | Pilus assembly protein PilW | 266 | WP_010327532.1 | *Acinetobacter lwoffii* | 99% |
| AAU60_13120 | Hypothetical protein | 767 | WP_010327535.1 | *Acinetobacter lwoffii* | 99% |
| AAU60_13125 | Protein CsuC | 234 | WP_053578792.1 | *Acinetobacter* sp. TTH0-4 | 79% |
| AAU60_13170 | Secretion protein HylD | 444 | WP_029575116.1 | *Acinetobacter lwoffii* | 98% |
| AAU60_13175 | Macrolide transporter | 663 | EXX02105.1 | *Acinetobacter baumannii* 44327_5 | 90% |
| AAU60_13180 | RND transporter | 469 | WP_010327545.1 | *Acinetobacter lwoffii* | 99% |
| AAU60_13800 | Mammalian cell entry protein | 224 | WP_010111868.1 | *Acinetobacter* sp. P8-3-8 | 79% |
| AAU60_13935 | Membrane protein | 699 | WP_050796980.1 | *Acinetobacter lwoffii* | 99% |
| AAU60_14030 | Pilus assembly protein PilE | 152 | WP_054580915.1 | *Acinetobacter* sp. 114 | 56% |
| AAU60_14035 | Pilin | 157 | WP_054580914.1 | *Acinetobacter* sp. 114 | 61% |
| AAU60_14040 | Pilus assembly protein PilY | 1302 | WP_054580913.1 | *Acinetobacter* sp. 114 | 56% |
| AAU60_14050 | Hypothetical | 311 | WP_054580911.1 | *Acinetobacter* sp. 114 | 60% |
| AAU60_14060 | Fimbrial biogenesis protein FimU | 153 | WP_016658474.1 | *Acinetobacter indicus* | 60% |
| AAU60_14105 | Fimbrial protein | 142 | WP_004678762.1 | *Acinetobacter parvus* | 81% |
| AAU60_14155 | Membrane protein | 687 | WP_004650599.1 | *Acinetobacter bohemicus* | 70% |
| AAU60_14250 | Hypothetical protein | 264 | WP_010327662.1 | *Acinetobacter lwoffii* | 96% |
| AAU60_14255 | ABC transporter ATP-binding protein | 553 | WP_049044347.1 | *Acinetobacter baumannii* | 99% |
| AAU60_14970 | Hypothetical protein | 256 | WP_053579241.1 | *Acinetobacter* sp. TTH0-4 | 74% |
| AAU60_15185 | ABC transporter substrate-binding protein | 261 | WP_044738836.1 | *Bacillus mycoides* | 92% |
| AAU60_15305 | RND transporter | 366 | WP_004649698.1 | *Acinetobacter bohemicus* | 78% |
| AAU60_15375 | Repeat protein | 2004 | WP_026441016.1 | *Acinetobacter tandoii* | 88% |
| AAU60_15415 | Major facilitator transporter | 452 | WP_044738814.1 | *Bacillus mycoides* | 92% |
| AAU60_15660 | Copper resistance protein CopB | 306 | WP_035269996.1 | *Acinetobacter sp. Ver3* | 98% |
| AAU60_15825 | MFS transporter | 381 | WP_004780342.1 | *Acinetobacter* sp. NIPH 899 | 75% |
| AAU60_15880 | ABC transporter ATP-binding protein | 646 | WP_004871608.1 | *Acinetobacter gerneri* | 87% |
| AAU60_16320 | Copper resistance protein CopB | 306 | WP_035269996.1 | *Acinetobacter* sp. Ver3 | 98% |
